# A Robust Complex *α*-Sigmoid Affine Projection Algorithm Under Non-Gaussian Noise

**DOI:** 10.3390/s26030961

**Published:** 2026-02-02

**Authors:** Yaowei Guo, Bin Guo, Guobing Qian

**Affiliations:** College of Electronic and Information Engineering, Southwest University, Chongqing 400715, China; 15833413651@163.com (Y.G.); guob0310@163.com (B.G.)

**Keywords:** adaptive filtering algorithm, affine projection algorithm, non-Gaussian noise, beamforming

## Abstract

To address the performance degradation of traditional adaptive filtering algorithms in environments with correlated input signals and non-Gaussian noise, this paper proposes a complex-valued affine projection algorithm based on the α-Sigmoid cost function (α-CSAP). The algorithm leverages the nonlinear characteristics of the α-Sigmoid function and implicitly achieves variable step-size updates by introducing a normalization factor, which effectively suppresses impulsive noise interference and avoids matrix inversion, thereby reducing computational complexity. Theoretical analysis derives the steady-state mean square deviation (MSD) expression for the algorithm. Simulation results demonstrate that the proposed α-CSAP algorithm exhibits superior performance compared to traditional complex adaptive filtering algorithms in both system identification and beamforming application scenarios.

## 1. Introduction

As a core technology in the field of digital signal processing, adaptive filtering, which does not require prior knowledge of the statistical characteristics of signals and noise and can dynamically adjust parameters to adapt to time-varying environments, has become a key means for radar systems to cope with complex signal and interference conditions [1,2,3,4]. Conventional fixed filters exhibit limited performance when dealing with non-stationary, nonlinear signals or unknown interference, whereas adaptive filtering, through iterative optimization algorithms such as Least Mean Square (LMS), Recursive Least Squares (RLS), and particle filtering, enables efficient denoising, target detection, and parameter estimation, and is widely applicable in engineering scenarios such as radar and communications [5,6,7].

In radar signal processing, adaptive filtering plays an indispensable and central role. Radar echoes are often affected by non-Gaussian noise such as spherically invariant clutter and pulse interference, making it difficult for traditional detection methods to achieve a satisfactory balance between detection probability and false alarm rate [8,9]. Adaptive filtering can effectively address such complex signal environments by dynamically adjusting filter weights or structures. For example, the adaptive matched filter can maintain a constant false alarm rate under unknown clutter covariance matrices, significantly improving radar target detection performance [10]; optimized particle filtering can achieve high-precision prediction of highly dynamic target trajectories, effectively handling tracking problems in nonlinear and non-Gaussian scenarios [11]. At the signal and data processing level, the RLS algorithm, with its fast convergence characteristics, is particularly suitable for processing non-stationary radar signals [12]. Furthermore, the integration of adaptive filtering with techniques such as digital beamforming and data fusion further enhances the anti-interference capability and target resolution accuracy of radar systems [13].

As a central direction in modern signal processing and machine learning, complex-valued adaptive filtering is of broad application value compared to real-valued filtering techniques, owing to its inherent suitability for complex signals prevalent in digital communications, power systems, radar, and other fields [14]. Traditional algorithms, often based on the Mean Square Error (MSE) criterion, are simple to implement but tend to become inaccurate or diverge in non-Gaussian impulsive noise environments. Moreover, they typically neglect the statistical properties of non-circular signals, leading to information loss [15]. The integration of information-theoretic criteria has led to algorithms based on complex correntropy and generalized complex correntropy, significantly enhancing robustness against non-Gaussian noise [16]. However, challenges remain, including the insufficient flexibility of single-kernel functions, incomplete utilization of error probability distributions, performance degradation under noisy input–output models, and the inherent trade-off between convergence speed and steady-state error [17]. In response, researchers have pursued optimization through various means, such as employing widely linear models to capture non-circular characteristics, introducing mixed-kernel functions to enhance adaptability, and designing fixed-point iterative or recursive forms to reduce complexity [18]. These efforts aim to better adapt these algorithms to complex engineering scenarios. Complementary to complex adaptive filtering, beamforming is a key technology in array signal processing. It enhances desired signals and suppresses interference by controlling the phase and amplitude of signals received by multiple sensors/antennas, demonstrating significant advantages in wireless communications, acoustic processing, and radar detection. Its core value lies in utilizing spatial diversity to improve signal quality and transmission efficiency, giving rise to specialized applications such as distributed collaborative beamforming [19], microphone array speech enhancement, and broadband beamforming with fractal antenna arrays [20]. Innovations in hybrid architectures and unsupervised methods further extend its applicability [21]. As important branches of adaptive signal processing, both complex adaptive filtering and beamforming face common challenges regarding adaptability in complex environments and balancing performance with complexity. Their technological integration and collaborative optimization have become important research directions driven by practical engineering needs.

In applications such as system identification, echo cancellation, and signal prediction, the performance of traditional adaptive filtering algorithms is significantly affected when the input signal is correlated. Their core operation lies in adjusting filter coefficients in real time to adapt to dynamic environments [22]. The classic LMS algorithm is widely adopted due to its simplicity, but its convergence speed degrades notably under correlated inputs. RLS algorithms converge faster but face high computational complexity, making them less suitable for real-time requirements [23]. To balance convergence speed and computational complexity, Ozeki and Umeda first proposed the Affine Projection (AP) algorithm in 1984 [24]. By reusing past input data, it achieves faster convergence than LMS while maintaining lower complexity than RLS, representing a major breakthrough in adaptive filtering [25]. However, practical challenges such as impulsive noise, highly correlated inputs, and sparse system characteristics impose limitations on traditional AP algorithms. For example, in acoustic echo cancellation and hands-free vehicular communications, impulsive interference can degrade algorithm performance [26]. In sparse system identification, conventional AP algorithms fail to exploit sparsity, leading to inefficient convergence [27]. In massive data processing scenarios, indiscriminate data updates result in wasted computational resources. Consequently, extensive research has been dedicated to improving the AP algorithm. Various strategies have been developed to enhance its performance. Data-selective updating mechanisms have been introduced to reduce computational load while maintaining estimation accuracy. Robust AP algorithms based on M-estimation have been designed to improve resilience against impulsive noise [28]. Variants incorporating sparsity-inducing penalties have been proposed for sparse system identification [29]. Furthermore, approaches that avoid direct or indirect matrix inversion have been adopted to lower the computational complexity of AP-type algorithms [30]. Additional techniques, such as subband processing and variable step-size strategies, have also been employed to further optimize convergence behavior under correlated inputs [31]. These improved algorithms demonstrate advantages in applications like system identification, echo cancellation, and beamforming. Nonetheless, achieving an optimal balance among robustness, real-time performance, and accuracy in complex environments remains an open challenge. Furthermore, while most existing AP algorithms are confined to the real domain, their application in the complex domain is increasingly important due to the prevalence of complex signals in radar signal processing.

Recently, researchers proposed an adaptive projection algorithm based on an α-sigmoid cost function, demonstrating a superior performance when dealing with strongly correlated input signals and non-Gaussian noise environments [32]. This provides a new approach for radar echo cancellation and channel estimation. Building upon this, we propose a novel complex-valued adaptive filtering algorithm based on the α-sigmoid cost function. The steady-state MSD of the proposed algorithm is derived analytically. The performance of the proposed algorithm is compared with classical complex adaptive filtering algorithms in simulations of system identification and beamforming. The results confirm the effectiveness and robustness of the proposed algorithm. Furthermore, the proposed algorithm offers a new potential solution for beamforming applications.

The remainder of this paper is organized as follows: Section 2 introduces the proposed algorithm. Section 3 provides a theoretical analysis of the algorithm, including the derivation of its steady-state mean MSD expression and an analysis of its computational complexity. Section 4 verifies the theoretical analysis and provides simulation results to demonstrate the superiority and robustness of the proposed algorithm. Section 5 concludes the paper.

## 2. The Proposed α-CSAP Algorithm

**Figure 1:**
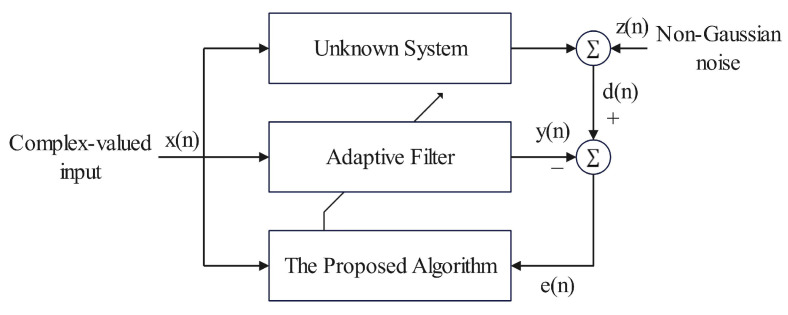
Block diagram for system identification.

As illustrated in Figure 1, the system identification model considered in this work is given by

(1) $$d\left(n\right)=x^{H}\left(n\right)w_{0}+z\left(n\right),$$

where $w_{0}\in C^{L_{w}\times 1}$ denotes the unknown system weight vector with $L_{w}$ being the filter length, z(n) is the complex-valued additive noise, and the input vector $x\left(n\right)\in C^{L_{w}\times 1}$ is defined as $x\left(n\right)={\left[x\left(n\right),x\left(n-1\right),\ldots ,x\left(n-L_{w}+1\right)\right]}^{T}$. Here, ${\left(\cdot \right)}^{H}$ denotes the Hermitian transpose. The output of the adaptive filter is expressed as $y\left(n\right)=x^{H}\left(n\right)w\left(n-1\right)$; here, $w\left(n-1\right)\in C^{L_{w}\times 1}$ is the adaptive weight vector at time index n−1. Accordingly, the instantaneous error signal is given by

(2) $$e\left(n\right)=d\left(n\right)-y\left(n\right)=d\left(n\right)-x^{H}\left(n\right)w\left(n-1\right).$$

In the AP algorithm framework, the projection order *Q* specifies the number of past input–error pairs used for coefficient adaptation. The stacked error vector is defined as $e\left(n\right)={\left[e\left(n\right),e\left(n-1\right),\ldots ,e\left(n-Q+1\right)\right]}^{T}\in C^{Q\times 1}$ and the input data matrix is constructed as $X\left(n\right)=\left[x\left(n\right),x\left(n-1\right),\ldots ,x\left(n-Q+1\right)\right]\in C^{L_{w}\times Q}$. In order to enhance the convergence rate of the algorithm and effectively mitigate the adverse effects of non-Gaussian noise on the adaptation process, the α-sigmoid function is employed as the cost function, as follows:

(3) $$J\left(n\right)=\sum_{i=0}^{Q-1}\frac{\alpha }{\alpha -1}{\left(1+\exp\left(-\frac{{\left|e\left(n-i\right)\right|}^{2}}{2\delta^{2}}\right)\right)}^{1-\frac{1}{\alpha }},$$

where the parameter α governs the steepness of the cost function, thereby determining the convergence speed of the algorithm: a larger α leads to a steeper function and faster convergence. The parameter δ adjusts the algorithm’s sensitivity to outliers. From a mathematical perspective, as the error e(n) increases, the curvature of the cost function gradually flattens, and its derivative with respect to the weights approaches zero, thereby achieving robust suppression of outliers.

Taking the Wirtinger derivative of J(n) with respect to the complex conjugate of the weight vector $w^{*}\left(n-1\right)$ yields

(4) $$\frac{\partial J(n)}{\partial w^{*}\left(n-1\right)}=-X\left(n\right)\eta \left(n\right)e^{*}\left(n\right),$$

where $\eta \left(n\right)\in R^{Q\times Q}$ is a diagonal weighting matrix defined as

(5) $$\eta \left(n\right)=\mathrm{diag}\{\eta_{1}\left(n\right),\ldots ,\eta_{Q}\left(n\right)\},$$

with

(6) $$\eta_{i}\left(n\right)=\frac{\exp\left(-\frac{{\left|e\left(n-i+1\right)\right|}^{2}}{2\delta^{2}}\right)}{\delta^{2}{\left(1+\exp\left(-\frac{{\left|e\left(n-i+1\right)\right|}^{2}}{2\delta^{2}}\right)\right)}^{1/\alpha }},\;i=1,2,\ldots ,Q.$$

Under the gradient descent framework, a preliminary update expression for the algorithm is formulated as follows:

(7) $$w\left(n\right)=w\left(n-1\right)+\mu X\left(n\right)\eta \left(n\right)e^{*}\left(n\right).$$

where μ is the step size.

In the derivation of the α SAP algorithm, the introduction of the normalization factor ${∥X\left(n\right)e\left(n\right)∥}_{2}$ is a key step to realize the $L_{2}$-norm constraint. The squared Euclidean norm of the weight update vector is expressed as follows:

(8) $${∥w\left(n\right)-w\left(n-1\right)∥}_{2}^{2}=\mu^{2}{∥X\left(n\right)\eta \left(n\right)e^{*}\left(n\right)∥}_{2}^{2}.$$

As can be observed from Equation (8), when the noise contains large outliers, the error e(n) will be significantly affected, which, in turn, substantially impacts the weight estimation and degrades the filter performance. The restriction of the filter update magnitude can effectively enhance the robustness of the filter. This can be expressed as follows:    

(9) $${∥w\left(n\right)-w\left(n-1\right)∥}_{2}^{2}\leq \varepsilon ,$$

where ε is a positive scalar. Taking the partial derivative of Equation (6), and according to Equation (8), the diagonal elements of the weighting matrix η(n) have an upper bound $\eta_{\max}=1/\left(\delta^{2}\cdot 2^{1/\alpha }\right)$, which leads to the following:

(10) $$\begin{array}{cc} \mu^{2}{∥X\left(n\right)\eta \left(n\right)e^{*}\left(n\right)∥}_{2}^{2}\; & \leq \mu^{2}\eta_{\max}^{2}{∥X\left(n\right)e\left(n\right)∥}_{2}^{2}\\ \frac{\mu^{2}{∥X\left(n\right)\eta \left(n\right)e^{*}\left(n\right)∥}_{2}^{2}}{{∥X\left(n\right)e\left(n\right)∥}_{2}^{2}}\; & \leq \mu^{2}\eta_{\max}^{2} \end{array}$$

At this point, by introducing a time-varying parameter ${∥X\left(n\right)e\left(n\right)∥}_{2}$ into the denominator of Equation (8) that shares the same order as the numerator, the upper bound for the squared norm of the squared Euclidean norm of the weight update vector is obtained, as follows:

(11) $${∥w\left(n\right)-w\left(n-1\right)∥}_{2}^{2}\leq \mu^{2}\eta_{\max}^{2}.$$

The update formula with constraints is expressed as follows:

(12) $$w\left(n\right)=w\left(n-1\right)+\mu \frac{X\left(n\right)\eta \left(n\right)e^{*}\left(n\right)}{{∥X\left(n\right)e\left(n\right)∥}_{2}}.$$

The proposed α-CSAP algorithm is summarized in Algorithm 1. The introduction of the normalization factor can be understood from an implicit variable step-size perspective. In scenarios with large errors, the effective step size $\mu /{∥X(n)e(n)∥}_{2}$ automatically decreases, thereby suppressing impulse noise interference and preventing filter divergence. In scenarios with small errors, the effective step size relatively increases, accelerating the convergence process. Moreover, traditional affine projection algorithms require the inversion or pseudo-inversion of the input matrix, which is prone to numerical instability due to ill-conditioned matrices. The normalization factor avoids matrix inversion operations, significantly reducing computational complexity.
**Algorithm 1:** The α-CSAP Algorithm**Require: ** x(n), d(n), $L_{w}$, *Q*, μ, α, $\delta^{2}$, *N***Ensure: ** w(n)1:Initialize $w\left(0\right)=0_{L_{w}\times 1}$2:**for** n=1 to *N* **do**3:    Construct: $x\left(n\right)={\left[x\left(n\right),x\left(n-1\right),\ldots ,x\left(n-L_{w}+1\right)\right]}^{T}$4:    Construct: $X\left(n\right)=\left[x(n),x(n-1),\ldots ,x(n-Q+1)\right]$5:    Compute: $y\left(n\right)=x^{H}\left(n\right)w\left(n-1\right)$6:    Compute: e(n)=d(n)−y(n)7:    Form: $e\left(n\right)={\left[e(n),e(n-1),\ldots ,e(n-Q+1)\right]}^{T}$8:    **for** i=1 to *Q* **do**9:        $\eta_{i}\left(n\right)=\frac{\exp\left(-\frac{{\left|e\left(n-i+1\right)\right|}^{2}}{2\delta^{2}}\right)}{\delta^{2}{\left[1+\exp\left(-\frac{{\left|e\left(n-i+1\right)\right|}^{2}}{2\delta^{2}}\right)\right]}^{1/\alpha }}$10:    **end for**11:    Construct: $\eta \left(n\right)=\mathrm{diag}\left\{\eta_{1}\left(n\right),\ldots ,\eta_{Q}\left(n\right)\right\}$12:    $w\left(n\right)=w\left(n-1\right)+\mu \cdot \frac{X\left(n\right)\eta \left(n\right)e^{*}\left(n\right)}{{∥X(n)e(n)∥}_{2}}$13:**end for**14:**return** w(n)

## 3. Theoretical Analysis

### 3.1. Unbiasedness in the Mean

This subsection employs a classical method from mean value analysis to conduct an approximate analysis of the mean unbiasedness of the proposed α-CSAP algorithm under reasonable assumptions.

Define the weight error vector as

(13) $$\tilde{w}\left(n\right)=w\left(n\right)-w_{0},$$

where $w_{0}$ denotes the unknown system weight vector. Substituting (12) into the above equation yields the update formula with constraints, which is expressed as:

(14) $$\tilde{w}\left(n\right)=\tilde{w}\left(n-1\right)+\mu \frac{X\left(n\right)\eta \left(n\right)e^{*}\left(n\right)}{{∥X\left(n\right)e\left(n\right)∥}_{2}}.$$

Under the affine projection framework with projection order *Q*, let the stacked desired signal vector be $d\left(n\right)={\left[d\left(n\right),d\left(n-1\right),\ldots ,d\left(n-Q+1\right)\right]}^{T}$ and the stacked noise vector be $z\left(n\right)={\left[z\left(n\right),z\left(n-1\right),\ldots ,z\left(n-Q+1\right)\right]}^{T}$. Thus, the stacked error vector can be expressed as

(15) $$e\left(n\right)=d\left(n\right)-X^{H}\left(n\right)w\left(n-1\right)=-X^{H}\left(n\right)\tilde{w}\left(n-1\right)+z\left(n\right).$$

Substituting the above expression into the normalized update rule of the proposed α-CSAP algorithm yields the following weight error recursion:

(16) $$\begin{array}{cc} \tilde{w}\left(n\right)= & \tilde{w}\left(n-1\right)-\mu \frac{X\left(n\right)\eta \left(n\right)X^{T}\left(n\right){\tilde{w}}^{*}\left(n-1\right)}{{∥X\left(n\right)e\left(n\right)∥}_{2}}\\ \; & +\mu \frac{X\left(n\right)\eta \left(n\right)z^{*}\left(n\right)}{{∥X\left(n\right)e\left(n\right)∥}_{2}}. \end{array}$$

To facilitate the mean analysis, the following assumptions are adopted.

- (A1) The input matrix X(n) is statistically independent of the weight error vector $\tilde{w}\left(n\right)$.
- (A2) In steady state, the diagonal weighting matrix η(n) varies slowly and can be approximated by
  (17) $$\eta \left(n\right)\approx \bar{g}I_{Q},$$
  where $\bar{g}=E\left\{\eta_{i}\left(n\right)\right\}$.
- (A3) ${∥X\left(n\right)e\left(n\right)∥}_{2}$ is weakly correlated with X(n) and $\tilde{w}\left(n-1\right)$, while z(n) is zero-mean and independent of both.

Under this assumption, it follows that $E\left\{\frac{X\left(n\right)\eta \left(n\right)z^{*}\left(n\right)}{{∥X\left(n\right)e\left(n\right)∥}_{2}}\right\}\approx 0$. Then, taking the expectation on both sides of (16) yields

(18) $$E\left\{\tilde{w}\left(n\right)\right\}\approx \left[I-\mu \bar{g}E\;\left\{\frac{X\left(n\right)X^{H}\left(n\right)}{{∥X\left(n\right)e\left(n\right)∥}_{2}}\right\}\right]E\left\{\tilde{w}\left(n-1\right)\right\}.$$

When the input signal is persistently exciting, the matrix inside the expectation is positive semi-definite. Therefore, for a sufficiently small step size μ, the only equilibrium point of (18) is

(19) $$\lim_{n\to \infty }E\left\{\tilde{w}\left(n\right)\right\}=0.$$

This result indicates that the proposed α-CSAP algorithm is unbiased in the mean.

### 3.2. Steady-State MSD Analysis

#### 3.2.1. Theoretical Value Derivation

In this subsection, the steady-state MSD performance of the proposed α-CSAP algorithm is analyzed. Due to the presence of nonlinear error weighting and normalization terms, the analysis is carried out using the energy conservation approach commonly adopted in the mean-square analysis of AP algorithms.

The MSD is defined as

(20) $$\mathrm{MSD}\left(n\right)≜E\left\{∥\tilde{w}{\left(n\right)∥}^{2}\right\}.$$

From the normalized update rule of the proposed algorithm, the weight error vector evolves as

(21) $$\tilde{w}\left(n\right)=\tilde{w}\left(n-1\right)-\mu K\left(n\right),$$

where

(22) $$K\left(n\right)=\frac{X\left(n\right)\eta \left(n\right)e^{*}\left(n\right)}{{∥X\left(n\right)e\left(n\right)∥}_{2}}.$$

Taking the squared Euclidean norm on both sides of (21), the expectation yields

(23) E∥w˜(n)∥2=E∥w˜(n−1)∥2−2μERew˜H(n−1)K(n)+μ2E∥K(n)∥2.

Under steady-state conditions, $E\left\{∥\tilde{w}{\left(n\right)∥}^{2}\right\}=E\left\{∥\tilde{w}{\left(n-1\right)∥}^{2}\right\}$, the MSD satisfies the following energy balance relation:

(24) 2μERew˜H(n−1)K(n)=μ2E∥K(n)∥2.

Define the a priori error vector as

(25) $$e_{a}\left(n\right)=X^{H}\left(n\right)\tilde{w}\left(n-1\right).$$

Then,

(26) $${\tilde{w}}^{H}\left(n-1\right)K\left(n\right)=\frac{e_{a}^{H}\left(n\right)\eta \left(n\right)e^{*}\left(n\right)}{{∥X\left(n\right)e\left(n\right)∥}_{2}}.$$

Since η(n) is a diagonal matrix with entries η(|e(n)|),…,η(|e(n−Q+1)|), the numerator in (26) can be expanded as

(27) $$e_{a}^{H}\left(n\right)\eta \left(n\right)e^{*}\left(n\right)=\sum_{i=0}^{Q-1}\eta \left(|e\left(n-i\right)|\right)\;e_{a}^{*}\left(n-i\right)\;e^{*}\left(n-i\right).$$

Following the standard mean-square analysis of APs, it is assumed that under steady-state and small-step-size conditions, the correlation between the numerator and the normalization factor in (26) is weak. Thus, the following separation approximation is adopted:

(28) $$E\left\{\frac{A}{B}\right\}\approx \frac{E\{A\}}{E\{B\}}.$$

Applying this approximation to (26), and noting that all *Q* components are statistically equivalent in steady state, yields

(29) ERew˜H(n−1)K(n)≈QEReη(|e|)ea∗e∗E{∥X(n)e(n)∥2}.

Define

(30) $$\varphi_{c}≜{E\{∥X\left(n\right)e\left(n\right)∥}_{2}\}.$$

Under steady-state conditions, the diagonal weighting function varies slowly and can be approximated by its average value, i.e.,

(31) $$\eta \left(|e|\right)\approx \bar{\eta },\;\bar{\eta }=E\{\eta (|e|)\}.$$

Therefore, the above expression reduces to

(32) ERew˜H(n−1)K(n)≈Qη¯φcEReea∗e∗.

For the complex-valued error $e=e_{a}+z$, where $e_{a}$ is a proper complex Gaussian random variable and *z* denotes Bernoulli–Gaussian impulsive noise, the weighting function operates on the magnitude of *e* only. By invoking Price’s theorem under the complex-valued setting, the following approximation holds:

(33) EReea∗e∗≈1πE{|ea|2}(1−Pr)E{|ea|2}+σα2.

Moreover, under steady-state conditions,

(34) $${∥X\left(n\right)e\left(n\right)∥}_{2}\approx \sqrt{Q\;E\{|e\left(n\right){|}^{2}\}}.$$

Combining the above results yields

(35) ERew˜H(n−1)K(n)≈η¯φcQE{|e(n)|2}·1πE{|ea(n)|2}(1−Pr)E{|ea(n)|2}+σα2.

The second-order term in (24) can be written as

(36) $$E\;\left\{{∥K\left(n\right)∥}^{2}\right\}=E\;\left[\frac{∥X\left(n\right)\eta \left(n\right)e^{*}{\left(n\right)∥}_{2}^{2}}{{∥X\left(n\right)e\left(n\right)∥}_{2}^{2}}\right].$$

Under steady-state and small-step-size conditions, this term can be approximated as

(37) $$E\;\left\{{∥K\left(n\right)∥}^{2}\right\}\approx {\bar{\eta }}^{2},$$

which is upper bounded by $\eta_{\max}^{2}$ due to the boundedness of the weighting function.

Using the relations

(38) $$E\{|e_{a}{\left(n\right)|}^{2}\}=\sigma_{x}^{2}{\;\mathrm{MSD},\;E\{|e\left(n\right)|}^{2}\}\approx \sigma_{x}^{2}\;\mathrm{MSD}+\sigma_{\alpha }^{2},$$

the steady-state MSD is finally obtained as

(39) $$\mathrm{MSD}=\frac{\mu \varphi_{c}\eta_{\max}\sigma_{\alpha }^{2}\left(\mu \varphi_{c}\eta_{\max}+\frac{2Q}{\sqrt{\pi }}\left(1-P_{r}\right)\right)}{\sigma_{x}^{2}\left[\frac{4Q^{2}}{\pi }{\left(1-P_{r}\right)}^{2}-\mu^{2}\varphi_{c}^{2}\eta_{\max}^{2}\right]}.$$

The above expression provides an accurate approximation of the steady-state MSD.

#### 3.2.2. Theoretical Validation

**Figure 2:**
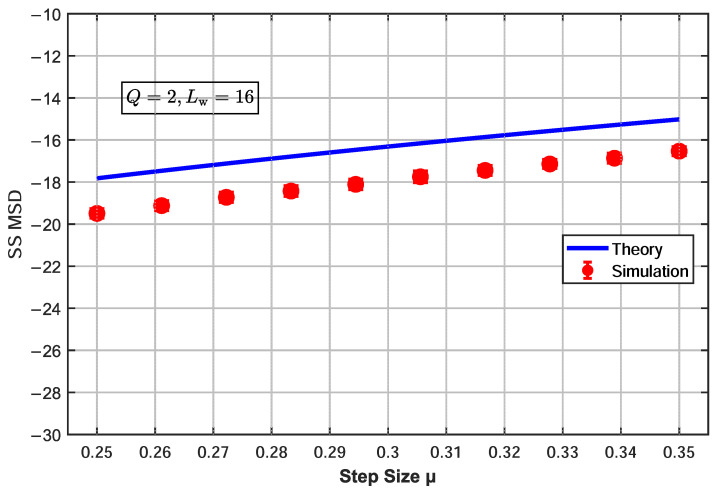
Effect of step-size parameter μ on steady-state MSD for α-CSAP algorithm (Q=2, $L_{w}=16$).

**Figure 3:**
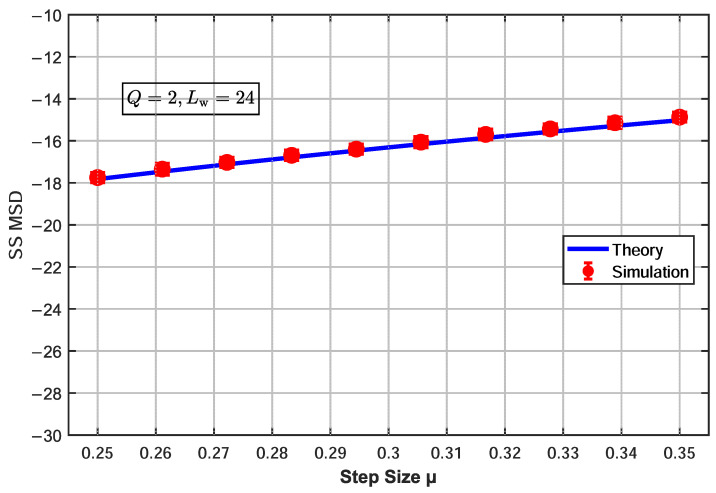
Effect of step-size parameter μ on steady-state MSD for α-CSAP algorithm (Q=2, $L_{w}=24$).

**Figure 4:**
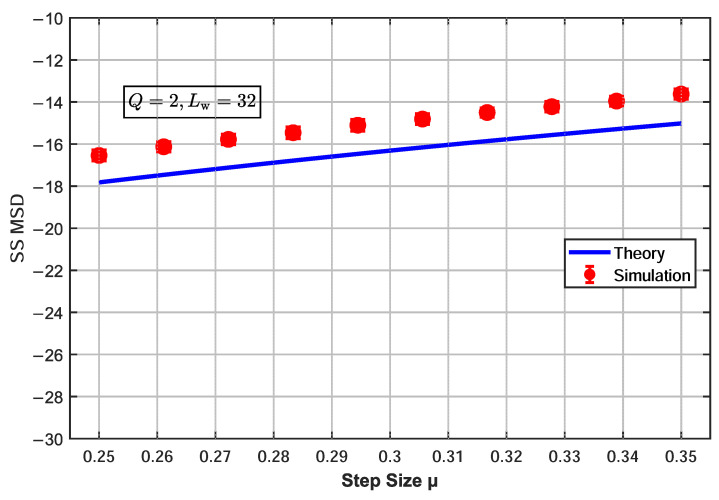
Effect of step-size parameter μ on steady-state MSD for α-CSAP algorithm (Q=2, $L_{w}=32$).

To verify the accuracy of the theoretical steady-state MSD model of the proposed α-CSAP algorithm, the steady-state MSD expression (39) derived from the theoretical analysis was validated through simulations under a projection order of Q=2, with the step-size parameter μ varying within the range μ∈[0.25,0.35]. The experimental setup was as follows: the filter length $L_{w}$ was set to three values, 16, 24, and 32, each matching the order of the unknown system; the number of iterations was N=1000, which was sufficient for the algorithm to reach steady state; and the results were averaged over 100 Monte Carlo trials. The input signal was a normalized complex-valued white noise, and the noise environment was modeled as Bernoulli–Gaussian mixture noise with an impulse probability of 0.02. Figure 2, Figure 3 and Figure 4 show the influence of the step-size parameter μ on the steady-state MSD for different filter lengths, with the projection order fixed at Q=2.

When $L_{w}=16$ (Figure 2), the theoretical MSD curve is slightly higher than the simulation data points, with an average error of 1.61 dB. As the filter length increases to $L_{w}=24$ (Figure 3), the theoretical and simulation results are in excellent agreement, verifying the accuracy of the derived expression under this configuration. When the filter length is further increased to $L_{w}=32$ (Figure 4), the predicted MSD values are slightly lower than the simulation results, with an average error of 1.35 dB. The slight deviations between the theoretical model and simulation results mainly originate from the necessary mathematical approximations adopted in the analysis, including the use of the separation assumption to handle the correlation of nonlinear terms, the steady-state averaging of the weighting function, and the approximate assumptions when applying Price’s theorem in the complex domain. Although these simplifications introduce systematic errors, they ensure the analytical tractability of the theoretical expressions. Experimental results demonstrate that the proposed theoretical model still accurately captures the overall trend of the steady-state MSD with respect to the step size, confirming the effectiveness of the analytical framework.

### 3.3. Computational Complexity Analysis

In this section, we conduct a comprehensive computational complexity analysis of the proposed α-CSAP algorithm. The operation counting baseline follows complex arithmetic rules: complex addition requires two real additions, and complex multiplication requires four real multiplications and two real additions. For a length-*n* complex inner product, 4n−2 real additions are required. The exponential function exp(·), approximated using a second-order Taylor expansion, is estimated to need five multiplications and three additions.

Table 1 details the computational complexity of the α-CSAP algorithm per iteration, where $L_{w}$ is the filter length and *Q* is the projection order.

Table 2 compares the computational complexity of different complex adaptive filtering algorithms. The algorithms under comparison include the Complex Least Mean Square (CLMS), the Complex Norm Least Mean Squares (CNLMS), the Complex Maximum Correntropy Criterion (CMCC), and the Complex Affine Projection (CAP) algorithms.

In terms of optimization potential, by employing fast recursive filtering (FRF) and an approximation of exp(·) requiring only three multiplications, the complexity of α-CSAP can be reduced to $6QL_{w}+3Q^{2}+8Q$ multiplications and $6QL_{w}+Q^{2}+6Q$ additions.

The proposed α-CSAP algorithm provides a good trade-off between computational complexity and performance. Although it requires more operations than LMS-type algorithms ($O(L_{w})$), it avoids the $O(Q^{3})$ matrix inversion of CAP and remains robust to impulsive noise and colored signals. In engineering applications, $L_{w}$ is often much larger than *Q*, so the complexity of the α-CSAP algorithm is dominated by the $O(QL_{w})$ term, making it suitable for real-time implementation.

## 4. Simulation

### 4.1. System Identification Application

This section evaluates the system identification performance of the proposed α-CSAP algorithm under various complex-valued non-Gaussian noise conditions, in comparison with several representative complex-valued adaptive filtering algorithms, including CLMS, CNLMS, CAP, and CMCC. The unknown system $w_{o}$ to be identified is represented by a complex impulse response vector of length $L_{w}=16$, whose real and imaginary parts are independently drawn from a Gaussian distribution with zero mean and a variance of 0.5. The input signal x(n) is a first-order complex autoregressive (AR(1)) process, generated by filtering complex-valued white Gaussian noise through a system function $H\left(z\right)=1/\left(1-0.9z^{-1}\right)$, resulting in strong temporal correlation. The projection order is set to Q=2. For a fair comparison, all algorithmic parameters are adjusted to achieve the same initial convergence speed. Simulation results are averaged over 100 Monte Carlo trials, and the MSD learning curve is adopted as the performance metric. The MSD at the *n*-th iteration is calculated as follows:

(40) $$\mathrm{MSD}\left(n\right)=10\log_{10}\left(∥w\left(n\right)-w_{0}{∥}^{2}\right).$$

To thoroughly assess the algorithm’s robustness, the following three distinct and representative models for complex-domain non-Gaussian noise are employed:

1. Impulsive Mixture Noise with Bernoulli–Gaussian Structure: The composite noise z(n) is defined as $z\left(n\right)=A\left(n\right)z_{1}\left(n\right)+B\left(n\right)z_{2}\left(n\right)$. Here, $z_{1}\left(n\right)∼\mathrm{CN}\left(0,\sigma_{g}^{2}\right)$ denotes the background complex Gaussian noise of variance $\sigma_{g}^{2}$. The impulsive element is represented by $z_{2}\left(n\right)∼\mathrm{CN}\left(0,\sigma_{i}^{2}/\mathrm{Pr}\right)$, having a larger variance $\sigma_{i}^{2}/\mathrm{Pr}$. A Bernoulli random variable A(n), distributed as Bernoulli(1−Pr) with Pr=0.001, governs the occurrence of impulses: A(n)=1 signifies no impulse, while its complement B(n)=1−A(n) indicates an impulse event. All variables A(n), $z_{1}\left(n\right)$, and $z_{2}\left(n\right)$ are mutually independent.
2. Contaminated Complex Gaussian Noise (CG Noise): This noise model is synthesized by summing two independent zero-mean complex Gaussian components. The background component has a variance of 0.008 for the real part and 0.002 for the imaginary part. An intermittent impulsive component, with a variance of 8 for the real part and 2 for the imaginary part, is superimposed with an occurrence probability of 0.01. The combined signal exhibits a heavy-tailed distribution characteristic due to the occasional high-power impulses.
3. Symmetric Complex α-Stable Noise (α-Stable Noise): This model adopts a complex-valued symmetric α-stable distribution, formulated by $z\left(n\right)=z_{R}\left(n\right)+jz_{I}\left(n\right)$. The real component $z_{R}\left(n\right)$ and imaginary component $z_{I}\left(n\right)$ are generated as independent and identically distributed real α-stable random variables, described by the distribution $z_{\alpha }\left(\alpha_{z},\beta_{z},\nu ,\xi \right)$. The configuration employs the following: characteristic exponent $\alpha_{z}=1.9$, symmetry parameter $\beta_{z}=0$, scale parameter ν=1, and location parameter ξ=0.

**Figure 5:**
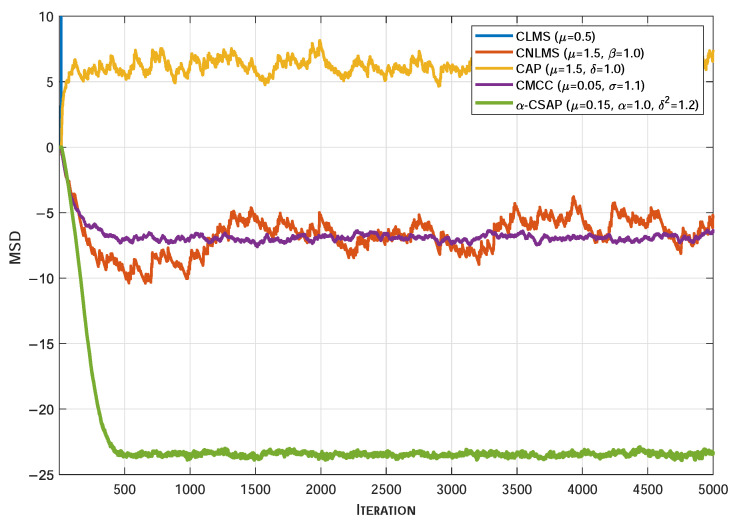
The MSD convergence curves of different algorithms under Bernoulli–Gaussian Impulsive Mixture Noise.

**Figure 6:**
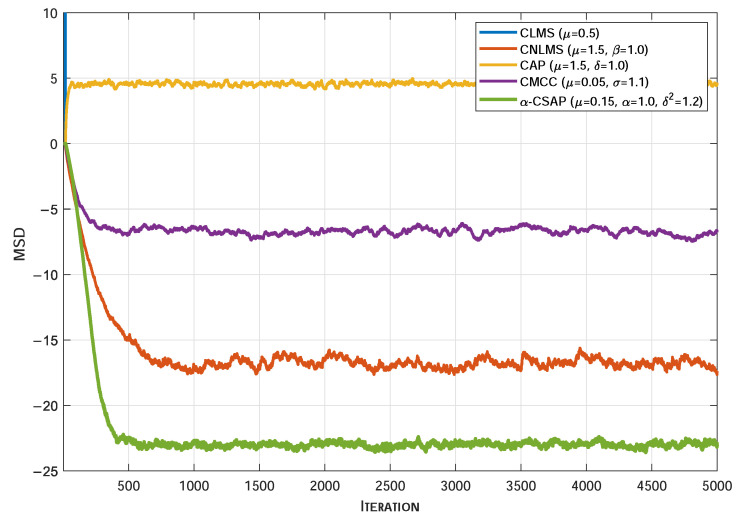
The MSD convergence curves of different algorithms under CG Noise.

**Figure 7:**
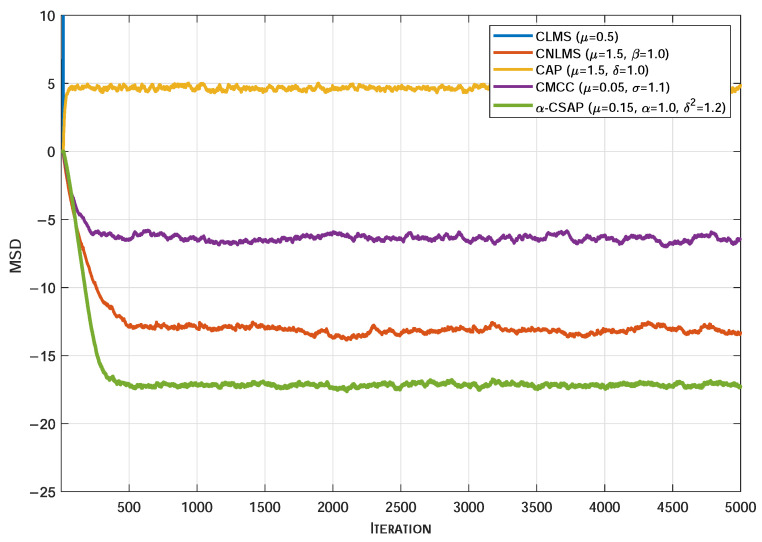
The MSD convergence curves of different algorithms under α-Stable Noise.

Figure 5, Figure 6 and Figure 7 illustrate the MSD convergence curves of various algorithms under three typical non-Gaussian noise environments. As can be seen, under the adverse conditions of strongly correlated input signals accompanied by non-Gaussian noise, the conventional CLMS algorithm exhibits a clear divergent trend. In contrast, the CNLMS algorithm effectively overcomes the divergence issue of LMS caused by step-size mismatch in non-stationary or impulsive signals through input signal power normalization of the step size, thereby maintaining system stability. However, the CAP algorithm suffers from a significantly degraded convergence performance under the influence of non-Gaussian noise. Furthermore, even when compared with robust algorithms specifically designed to suppress impulsive interference, the proposed α-CSAP algorithm still achieves the lowest steady-state MSD across all three noise scenarios. This is primarily attributed to the synergistic design of the α-sigmoid cost function and the $\ell_{2}$-norm constraint adopted in the α-CSAP algorithm, which endows it with stronger anti-interference capability. In summary, the comparative results demonstrate that the proposed α-CSAP algorithm exhibits superior robustness and a more accurate steady-state estimation performance when handling strongly correlated input signals and complex non-Gaussian noise.

### 4.2. Beamforming Application

This section evaluates the beamforming performance of the proposed α-CSAP algorithm in impulsive noise environments. A hexagonal sensor array composed of 91 elements is employed, with an array radius set to three times the operating wavelength and an inter-element spacing of approximately half a wavelength. The operating frequency is 8 GHz, corresponding to a free-space wavelength of λ=37.5 mm. Five independent narrowband signals with Quadrature Phase Shift Keying (QPSK) modulation are simulated, including one desired signal and four strong interferences. The direction of arrival (DOA) of the desired signal is at an azimuth of $90^{\circ }$ and an elevation of $45^{\circ }$. The DOAs of the four interfering signals are $\left(20^{\circ },45^{\circ }\right)$, $\left(62^{\circ },45^{\circ }\right)$, $\left(120^{\circ },45^{\circ }\right)$, and $\left(145^{\circ },45^{\circ }\right)$, respectively. Herein, the azimuth angle θ is defined clockwise with respect to the array normal, and the elevation angle φ denotes the angle between the incident signal and the array plane.

To simulate a realistic strong interference environment, high-intensity impulsive noise is introduced, which is modeled by a Bernoulli–Gaussian mixture distribution. The impulse occurrence probability is set to 0.05, and the variances of the corresponding Gaussian components are $\sigma_{1}^{2}=2$ and $\sigma_{2}^{2}=40$, respectively. The Signal-to-Noise Ratio (SNR) and Interference-to-Noise Ratio (INR) are set to 20 dB and 40 dB, respectively.

The core parameters of the α-CSAP algorithm are systematically tuned, as follows: the adaptive filter length $L_{w}$ equals the number of array elements (i.e., 91), the projection order is Q=4, and the step size is set to μ=0.0001. Furthermore, the characteristic exponent α and the scale parameter δ of the algorithm are configured as 1.5 and 16, respectively. This configuration significantly enhances the stability and robustness of the algorithm under impulsive noise. For a fair comparison, the step sizes of the conventional algorithms are also adjusted accordingly, as follows: $\mu_{\mathrm{CLMS}}=0.001$ for the CLMS algorithm, $\mu_{\mathrm{CAP}}=0.0005$ for the CAP algorithm, and $\mu_{\mathrm{CMCC}}=0.0003$ for the CMCC algorithm. This ensures that all algorithms exhibit similar initial convergence speeds under identical simulation conditions.

**Figure 8:**
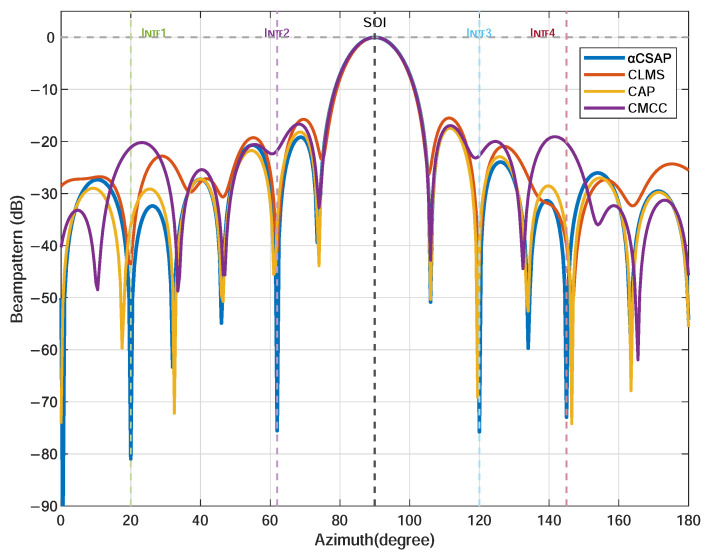
Comparison of beampatterns: CLMS, CAP, CMCC, and α-CSAP.

As shown in the beam pattern results in Figure 8, while maintaining the same mainlobe gain, the α-CSAP algorithm achieves deeper nulls at the four interference directions of $20^{\circ }$, $62^{\circ }$, $120^{\circ }$, and $145^{\circ }$, with null depths exceeding those of the other compared algorithms.

We consider the signal-to-interference-plus-noise ratio (SINR) as a metric to evaluate the performance of the beamforming system, which represents the ratio of the desired signal power to the interference-plus-noise power. As shown in Table 3, under the harsh condition of an input SINR as low as −26.30 dB, the α-CSAP algorithm elevates the output SINR to 34.72 dB, achieving a remarkable improvement of 61.02 dB. This performance enhancement significantly surpasses that of the other compared algorithms. These results fully demonstrate the superior capability of the α-CSAP algorithm in interference suppression and overall beamforming performance.

## 5. Conclusions

This paper proposes a robust complex α-CSAP suitable for non-Gaussian noise environments. By extending the affine projection algorithm based on the α-Sigmoid cost function to the complex domain, the algorithm enhances robustness against non-Gaussian noise. The computational complexity is reduced by employing a normalization factor to avoid matrix inversion. In two typical application scenarios, system identification and beamforming, the proposed α-CSAP algorithm demonstrates superior convergence characteristics and lower steady-state error compared to classical complex adaptive filtering algorithms such as CLMS, CAP, and CMCC. The results validate its effectiveness and engineering potential in complex-domain signal processing.

## Figures and Tables

**Table 1:** Computational complexity of α-CSAP algorithm (per iteration).

Step	×	+	exp(·)	÷
Initialization: w(n)=0				
1. $e\left(n\right)=d\left(n\right)-X^{H}\left(n\right)w\left(n-1\right)$	$4QL_{w}$	$4QL_{w}-2Q$	0	0
2. $|e_{k}{\left(n\right)|}^{2}$, k=1,…,Q	2Q	*Q*	0	0
3. $\exp(-|e_{k}\left(n\right){|}^{2}/\left(2\delta^{2}\right))$	5Q	3Q	*Q*	0
4. $\eta_{i}\left(n\right)=\frac{\exp(\cdot )}{\delta^{2}{\left(1+\exp\left(\cdot \right)\right)}^{1/\alpha }}$	4Q	2Q	0	*Q*
5. X(n)e(n)	$4QL_{w}$	$4QL_{w}-2Q$	0	0
6. $X\left(n\right)\eta \left(n\right)e^{*}\left(n\right)$	$4Q^{2}+4QL_{w}$	$2Q^{2}+4QL_{w}-2Q$	0	0
7. ${∥X\left(n\right)e\left(n\right)∥}_{2}$	2Q+4	Q+1	0	0
8. $w\left(n\right)=w\left(n-1\right)+\mu \frac{X\left(n\right)\eta \left(n\right)e^{*}\left(n\right)}{{∥X\left(n\right)e\left(n\right)∥}_{2}}$	$4QL_{w}+2$	$4QL_{w}+1$	0	1
Total	$8QL_{w}+4Q^{2}+13Q+6$	$8QL_{w}+2Q^{2}+12Q-1$	*Q*	Q+1

**Table 2:** Computational Comparison of Different Complex Adaptive Algorithms.

Algorithm	×	+	exp(·)	÷
CLMS	$8L_{w}+4$	$8L_{w}-2$	0	0
CNLMS	$12L_{w}+8$	$12L_{w}-5$	0	1
CMCC	$8L_{w}+26$	$8L_{w}+12$	1	0
CAP	$8QL_{w}+4Q^{2}+4Q^{3}$	$8QL_{w}+4Q^{2}+4Q^{3}-6Q$	0	0
α-CSAP	$8QL_{w}+4Q^{2}+13Q+6$	$8QL_{w}+2Q^{2}+12Q-1$	*Q*	Q+1

**Table 3:** Beampattern gain values and SINR performance comparison in impulsive noise environment.

Algorithm	Final SINR (dB)	SINR Improvement (dB)	Interference Direction (dB)			
			20°	62°	120°	145°
α-CSAP	34.72	+61.02	−80.95	−75.53	−75.76	−72.94
CLMS	18.72	+45.02	−43.52	−41.05	−43.10	−39.92
CAP	11.44	+37.74	−36.14	−35.33	−39.49	−38.04
CMCC	12.27	+38.57	−21.15	−21.73	−22.72	−20.28

Input SINR = −26.30 dB. The beampattern gain in the desired direction (90°) is normalized to 0.00 dB.

## Data Availability

The data presented in this study are available on request from the corresponding author.

## Abbreviations

The following abbreviations are used in this manuscript:

α-CSAP	Complex α-Sigmoid Affine Projection
MSD	Mean Square Deviation
LMS	Least Mean Square
RLS	Recursive Least Squares
MSE	Mean Square Error
AP	Affine Projection
CLMS	Complex Least Mean Square
CNLMS	Complex Norm Least Mean Squares
CAP	Complex Affine Projection
CMCC	Complex Maximum Correntropy Criterion
CG Noise	Contaminated Complex Gaussian Noise
α-Stable Noise	Symmetric Complex α-Stable Noise
QPSK	Quadrature Phase Shift Keying
DOA	Direction Of Arrival
INR	Interference-to-Noise Ratio
SNR	Signal-to-Noise Ratio
INR	Interference-to-Noise Ratio
SINR	Signal-to-Interference-plus-Noise Ratio
